# Pharmacometric modeling of opioid disposition in pregnancy: a systematic review of PopPK and PBPK approaches, model limitations, and future directions

**DOI:** 10.3389/fphar.2026.1789714

**Published:** 2026-04-20

**Authors:** Syed Saoud Zaidi, Mohammad Yaseen Abbasi, Sruthi Ganapaneni, Sara K. Quinney

**Affiliations:** 1 Division of Clinical Pharmacology, Department of Medicine, Indiana University School of Medicine, Indianapolis, IN, United States; 2 Creighton University School of Medicine – Phoenix, Phoenix, AZ, United States; 3 Department of Obstetrics and Gynecology, Indiana University School of Medicine, Indianapolis, IN, United States

**Keywords:** Maternal–fetal pharmacology, opioid use disorder (OUD), opioids, physiologically based pharmacokinetic (PBPK) modeling, population pharmacokinetics (PopPK), pregnancy pharmacokinetics

## Abstract

Physiological changes during pregnancy substantially alter opioid pharmacokinetics, yet clinical pharmacokinetic data in pregnant populations remain limited. Pharmacometric modeling using population pharmacokinetic (PopPK) and physiologically based pharmacokinetic (PBPK) approaches offers a quantitative framework to characterize these changes and to estimate maternal and fetal drug exposure. Despite increasing application of these methods, the methodological quality, reproducibility, and clinical relevance of existing opioid models in pregnancy have not been systematically evaluated. This systematic review aimed to critically appraise published PopPK and PBPK models of opioid medications in pregnancy, identify recurring structural and methodological features, and outline priorities for improving model development and reporting. A structured PubMed search (database inception to September 2025) identified studies modeling opioid disposition in pregnant individuals using PopPK or PBPK frameworks. Data on model structure, parameterization, assumptions, evaluation strategies, and reported limitations were extracted and synthesized. Ten studies met inclusion criteria, with fentanyl having the most studies. PopPK analyses consistently identified pregnancy as a significant covariate associated with increased clearance. PBPK models showed substantial heterogeneity in physiological detail, ranging from simplified fetoplacental compartments to permeability-limited placenta representations and multi-compartment fetal systems. Reproducibility emerged as a concern, with at least one PBPK model unable to be independently replicated due to incomplete reporting. Across studies, common gaps included limited verification datasets, incomplete representation of gestational physiology, and reliance on single-time-point umbilical cord concentrations at delivery. Overall, pharmacometric modeling provides valuable mechanistic insight into opioid disposition during pregnancy, but its translational impact is constrained by inconsistent reporting practices, sparse empirical data, and limited incorporation of fetal and neonatal exposure. Future progress will be accelerated by standardized documentation, improved pregnancy-specific physiological data, integration of genetic and developmental variability, and closer linkage between pharmacokinetics and clinically relevant outcomes to support model-informed perinatal opioid therapy.

## 1 Introduction

Opioid administration during pregnancy, whether for the alleviation of pain or the treatment of Opioid Use Disorder (OUD), poses a considerable clinical dilemma (Whittaker et al., 2026). Conventional dosing protocols, which are typically formulated for non-pregnant adult populations, frequently fail to account for the physiological changes that occur during pregnancy (Little, 1999). Physiological changes during pregnancy may significantly influence pharmacokinetics (PK), including plasma volume, plasma protein abundance, and drug-metabolizing enzyme and transporter activity. These alterations may result in either underdosing or excessive exposure, with clinical consequences that differ by indication. For pain management, underdosing may lead to inadequate analgesia, whereas for OUD treatment, insufficient dosing places the mother at risk of withdrawal symptoms and relapse. Conversely, excessive exposure may lead to adverse effects for both the mother and the developing fetus, including neonatal opioid withdrawal syndrome (Silva et al., 2022). For decades, ethical considerations have limited the involvement of pregnant individuals in clinical research, creating a substantial gap in the existing evidence (Costantine, 2014). As a result, clinicians have often had to make dosing decisions without adequate empirical evidence, a gap that has pushed pharmacometric modeling into a central role. This framework allows investigators to draw on sparse clinical or *in vitro* data, together with basic physiological principles, to approximate how a drug behaves in the pregnant population (Shiu and Ensom, 2012). Two main strategies are used in pharmacometric modeling. Population pharmacokinetics (PopPK) takes a top-down, data-driven route using nonlinear mixed effect analyses of clinical concentration-time profiles of medication exposure to characterize variability within a population and to pinpoint patient factors associated with that variability (Berezowska et al., 2024). By contrast, physiologically based pharmacokinetic (PBPK) models follow a bottom-up, mechanistic logic, assembling a virtual organism composed of physiologically defined organ-level compartments combined with mechanistic understanding of a drug’s disposition. A side-by-side comparison of PopPK and PBPK modeling is provided in Table 1. It uses a drug’s physicochemical attributes and established physiological information to anticipate its movement through the body (Avram, 2020).

**TABLE 1:** Comparison of PBPK and PopPK methods.

Feature	Population PK (PopPK)	Physiologically-based PK (PBPK)
Core Principle	“Top-Down”: Empirical, statistical approach to describe observed clinical data and quantify sources of variability	“Bottom-Up”: Mechanistic approach to predict drug disposition from first principles of physiology and biochemistry
Data Input	Clinical PK data (often sparse) from pregnant individuals; patient covariates (e.g., gestational age, weight)	Drug physicochemical properties; *in vitro* metabolism/transport data; system parameters (organ volumes, blood flows, enzyme abundance)
Handling of Pregnancy	Empirical: Pregnancy, trimester, or gestational age is treated as a covariate that statistically influences PK parameters (e.g., clearance)	Mechanistic: Gestational changes (e.g., increased plasma volume, enzyme induction) are explicitly modeled as time-varying physiological parameters
Fetal Exposure	Cannot predict *a priori*. Requires direct measurement of umbilical cord blood for inclusion in the model	Uniquely capable of predicting placental transfer and fetal tissue concentrations without direct measurement
Primary Strength	Quantifying the magnitude of pregnancy’s effect and other sources of inter-individual variability from real-world clinical data	Predicting PK changes across gestation in unstudied scenarios and providing a mechanistic understanding of why those changes occur

This review aims to systematically examine the literature on pharmacometric PopPK and PBPK modeling approaches used to study opioid medications during pregnancy. Beyond descriptive synthesis, we critically assess how these models are constructed, identify recurring structural and methodological limitations, and discuss concrete steps to improve future model development and promote more rigorous and transparent best practices.

## 2 Methods

This systematic review assessed scientific literature for published PopPK and PBPK models of opioids in pregnancy, including any gestational age, during delivery, and immediately postpartum. Opioids were considered if indicated for pain management or treatment of OUD. For each pertinent publication, we extracted data regarding model development, including the software platform, model architecture, data sources, and principal assumptions, as well as identifying model limitations encompassing data deficiencies, methodological inadequacies, and uncertainties associated with parameter estimation.

A literature search was conducted in PubMed®, covering the period from database inception through September 2025. Searches were performed using the default ‘All Fields’ mode, which queries titles, abstracts, MeSH terms, and other indexed fields. The search strategy combined opioid-specific terms with pregnancy-related terminology and PK modeling descriptors. For each opioid, the drug name was paired with PK terms (“pharmacokin*”, “PBPK”, “PKPD”, or “concentration”) and pregnancy-related terms (“pregnancy”, “obstet*”, “matern*”, “mother”, or “lactat*”). This approach facilitated the identification of PBPK studies concerning methadone, buprenorphine, and fentanyl; PopPK analyses related to oxycodone during labor and sufentanil; and placenta-transfer investigations pertinent to morphine. Only articles published in the English language were deemed eligible, and the reference lists of pertinent studies were scrutinized manually to uncover additional relevant publications.

Studies were deemed eligible for inclusion if they either developed or applied a PopPK or PBPK model concentrated on an opioid pharmacotherapeutic agent, and involved a pregnant human cohort, whether focusing solely on maternal subjects or encompassing maternal–fetal interactions. Studies were excluded from consideration if they were exclusively conducted in settings involving non-pregnant populations or animal models, if they constituted review or commentary articles devoid of original modeling, or if they were reliant exclusively on noncompartmental analyses without a substantive PopPK or PBPK modeling framework.

A total of 2,860 records were identified across 26 opioid agents related to human pregnancy. Primary research articles published between 2006 and 2025 were screened for eligibility. Following title, abstract, and full-text review, 10 studies met the predefined inclusion criteria. Two independent reviewers conducted all screening steps, with disagreements resolved by consensus. Data extraction was performed independently by both reviewers using structured evidence tables. For PopPK studies, extracted information included study design, participant demographics, gestational age, dosing regimens, modeling software and estimation methods, model structure, covariate relationships, model evaluation approaches, and reported dosing simulations. For PBPK studies, extracted elements included the modeling platform and version, model scope (full or minimal), representation of placental and fetal compartments, predictive performance metrics when reported (including fold error), model development strategy, verification against independent datasets, sensitivity analyses, and reported applications such as maternal or fetal exposure prediction or assessment of drug–drug interactions. In addition to descriptive data extraction, identified PopPK and PBPK models were examined with respect to their transparency of reporting, physiological plausibility of structural and parameter assumptions, and approaches to model evaluation and predictive performance. Assessment focused on features explicitly reported by the original authors, including model structure, handling of pregnancy-related physiological changes, data sources used for calibration or validation, and the use of quantitative performance metrics, where available. No formal quality scoring system was applied.

## 3 Results

Among the 26 opioid medications included in the search, PopPK or PBPK models were identified for only morphine, codeine, buprenorphine, fentanyl, oxycodone, methadone, and sufentanil (Table 2; Table 3). Published pharmacometric modeling studies of opioid therapy during pregnancy vary substantially across compounds. Among the opioids identified in this review, buprenorphine was the most extensively characterized using PBPK modeling approaches, with multiple maternal–fetal models explicitly incorporating placental transfer and fetal exposure (Abduljalil et al., 2020; Zhang et al., 2018; Kalluri et al., 2017). Fentanyl was evaluated in three pharmacometric studies spanning both PBPK and population pharmacokinetic (PopPK) frameworks, addressing distinct questions related to drug–drug interactions, maternal–fetal transfer, and neonatal target-site exposure (Alsmadi, 2023; Cambic et al., 2014; Shum et al., 2021). Methadone was examined in a limited number of PBPK studies; however, no pregnancy-specific PopPK models were identified despite its widespread and longstanding clinical use (Badhan and Gittins, 2021; Ke et al., 2014a). In comparison, modeling efforts for oxycodone, codeine, and sufentanil were sparse and generally narrower in scope, typically consisting of single PopPK or PBPK analyses (Badaoui et al., 2021; Kokki et al., 2012; Nie et al., 2025). Additional details of each study are provided in the Supplementary Material.

**TABLE 2:** Opioid drugs for which models are available in pregnancy.

Drug	PBPK studies	PopPK studies	Total
Buprenorphine	2	0	2
Methadone	2	0	2
Fentanyl	2	1	3
Oxycodone	0	1	1
Codeine & Morphine	1	0	1
Sufentanil	0	1	1

**TABLE 3:** Summary of pharmacokinetic models of opioids in pregnancy.

Drug name	References	Model type/Platform	Validation criteria	Limitations of the model	Primary finding
Buprenorphine	Zhang et al. (2018)	PBPK (Simcyp v15.1)	AUC, Cavg, Cmax: within ±25% of observed means except Cavg in T3 (−26.3%)	Sublingual route implementation poorly documented → reproducibility concerns; underpredicted clearance in T2/T3 suggesting need to better characterize CYP3A4/UGT induction^a^ ^,^ ^b^ ^,^ ^c^ ^,^ ^f^	Pregnancy substantially reduces buprenorphine systemic exposure (∼50% in T3) due to increased hepatic clearance and volume of distribution, suggesting clinical need for increased dosing frequency
Buprenorphine	van Hoogdalem et al. (2024)	PBPK (Simcyp v21.0)	Maternal AUC0-12h, CL, Cmax within 1.25-fold; fetal geometric mean predicted/observed cord blood = 1.15; 95.2% within 2-fold error	Empirical global scalar (0.26819) for Kp values lacking experimental validation; accurate fetal prediction required post hoc adjustment of maternal absorption; assumes pH-driven absorption reduction applies identically across formulations/doses^a^ ^,^ ^b^ ^,^ ^f^	Fetal exposure increases throughout gestation; accurate prediction of fetal concentrations required post hoc optimization of maternal sublingual absorption to account for high inter-individual variability and pregnancy-induced reductions in salivary pH
Methadone	Ke et al. (2014a)	PBPK (Simcyp v12.1); Maternal PBPK model with single placental-fetal compartment	Pred/Obs AUC Cmax within 0.8–1.25 in T2/T3	CYP2B6 induction magnitude estimated from *in vitro* mRNA data (IVIVE), not calibrated against *in vivo* reference; validation using methadone potentially insensitive due to multiple clearance pathways (CYP2B6, 3A, 2C19, renal); unable to assess fetal disposition^b^ ^,^ ^f^	Model successfully predicted methadone PK in T2 (ratio 0.8–1.25); T3 predictions less robust, slightly underpredicting concentrations (AUC ratio 0.79)
Methadone (R/S stereoisomers)	Badhan and Gittins (2021)	PBPK (Simcyp v19); Full mechanistic maternal-fetal PBPK model	Verified against observed R- and S-methadone concentrations in non-pregnant subjects; maternal gestational validation relied on comparison to observed racemic methadone concentrations	Validation of each stereoisomer against total racemic methadone concentration only^a^ ^,^ ^b^ ^,^ ^c^ ^,^ ^f^	At standard 90 mg daily dose, 75% (R-methadone) and 94% (S-methadone) of women had subtherapeutic trough levels (80 ng/mL) by week 40; 29%–45% of fetuses had levels below threshold (60 ng/mL) to potentially limit NAS
Fentanyl	Shum et al. (2021)	MATLAB; Full maternal-fetal PBPK (14-comp + 7-comp)	Most predictions AAFE ≤2; matched maternal and umbilical cord data	Major uncertainty in fetal/umbilical unbound fraction (fu); assumptions in epidural compartment scaling; model code not provided limiting reproducibility^a^ ^,^ ^b^ ^,^ ^c^ ^,^ ^f^	Standard epidural dosing predicted to be safe; simulations of high-dose IV fentanyl (mimicking illicit use) predicted fetal concentrations capable of causing respiratory depression
Fentanyl	Cambic et al. (2014)	PK simulations using SAAM II software; 3-comp model plus epidural absorption	Maximum plasma fentanyl concentrations: 1.8 ng/mL (control) and 6.1 ng/mL (ritonavir simulations) based on highest standard dosing regimen	Relied on parameters derived from non-pregnant population; did not account for pregnancy-induced physiological changes^d^ ^,^ ^e^ ^,^ ^f^	Although ritonavir (potent CYP3A4 inhibitor) increased maternal plasma fentanyl concentrations, levels remained well below respiratory depression threshold (6.1 ng/mL) even during maximal patient-controlled epidural analgesia dosing
Fentanyl	Alsmadi (2023)	PK-Sim/MOBI 9; Full maternal-fetal PBPK model and full newborn PBPK model	Simulations deemed adequate if AFE and PK parameter ratios fell within 0.5–2.0	Adult metabolic values (Vmax, Km) and tissue partition coefficients applied to mothers and newborns; adult respiratory depression thresholds used for infants due to lack of specific data^a^ ^,^ ^d^ ^,^ ^f^	Near-perfect correlation identified between fentanyl AUC in newborn saliva and brain extracellular fluid (bECF), establishing saliva as valid non-invasive surrogate for brain exposure
Oxycodone	Kokki et al. (2012)	Population PK using NONMEM FOCE-I; Two-compartment model with first-order elimination	Bootstrap (200/500) and numerical predictive check (n = 200)	Small sample size (n = 15); limited mechanistic insight^a^ ^,^ ^b^ ^,^ ^c^ ^,^ ^e^ ^,^ ^f^	Maternal elimination half-life (median 2.6 h) significantly shorter than in non-pregnant women (3.8 h); oxycodone crossed placenta freely with umbilical concentrations similar to maternal concentrations at birth
Codeine/Morphine	Badaoui et al. (2021)	PBPK (Simcyp v19.1); Full maternal and fetal PBPK models	Simulated/observed MFE ∼1.2 (codeine) and ∼1.15 (morphine) for AUC/Cmax fell within ≤2-fold acceptance criteria	Virtual populations only; no pregnant human genotype-stratified validation; Single dose simulation only(for lower back pain opioid may be taken multiple dose; fetal genotype mismatch ignored^a^ ^,^ ^f^	Maternal CYP2D6 phenotype significantly determined fetal exposure; ultra-rapid metabolizer (UM) cohort showed >1.65-fold increase in fetal morphine AUC compared to extensive metabolizers (EM)
Sufentanil	Nie et al. (2025)	PopPK using NONMEM v7.4.0; 2-compartment model (maternal + umbilical compartments)	Model fit maternal venous and umbilical concentration vs. time data; VPCs reported	Concomitant ropivacaine may confound; very low intercompartmental clearance estimate (CL2 = 0.0134 L/h, representing 0.01% of maternal clearance)^a^ ^,^ ^b^ ^,^ ^c^ ^,^ ^e^ ^,^ ^f^	Model predicts slow decline in umbilical cord concentration after epidural infusion discontinuation, suggesting sufentanil sequestered in placenta due to high lipophilicity and released slowly back into fetal circulation

^a^ Sparse fetal/neonatal sampling: Single umbilical cord sample or limited neonatal blood draws restricting precision of fetal parameter estimation.

^b^ No active metabolite kinetics: Pharmacologically active metabolites (e.g., norbuprenorphine, EDDP, norfentanyl, oxymorphone, M3G/M6G) not incorporated into the model.

^c^ Fetal unbound fraction (fu) uncertainty: Fetal protein binding assumed from adult values or not directly measured.

^d^ Non-pregnant PK, parameters applied: Model relied on pharmacokinetic parameters derived from non-pregnant populations without pregnancy-specific adjustments.

^e^ Limited covariate analysis: Patient-specific factors (e.g., CYP, genotype, body weight, gestational age) influencing drug disposition not fully characterized.

^f^ No clinical outcome correlation: Model predictions not validated against neonatal clinical outcomes (e.g., NAS/NOWS, incidence or severity).

Abbreviations: AAFE, absolute average fold error; AFE, average fold error; AUC, area under the concentration-time curve; bECF, brain extracellular fluid; Cavg, average concentration; CL, clearance; Cmax, maximum concentration; comp, compartment; EDDP, 2-ethylidene-1, 5-dimethyl-3, 3-diphenylpyrrolidine; EM, extensive metabolizer; FOCE-I, first-order conditional estimation with interaction; fu, unbound fraction; IV, intravenous; IVIVE, in vitro-in vivo extrapolation; Km, Michaelis-Menten constant; Kp, tissue-to-plasma partition coefficient; M3G, morphine-3-glucuronide; M6G, morphine-6-glucuronide; MFE, mean fold error; NAS, neonatal abstinence syndrome; NOWS, neonatal opioid withdrawal syndrome; PBPK, physiologically based pharmacokinetic; PK, pharmacokinetic; PopPK, population pharmacokinetic; Pred/Obs, predicted/observed; T2, second trimester; T3, third trimester; UM, ultra-rapid metabolizer; Vmax, maximum velocity; VPC, visual predictive check.

### 3.1 Buprenorphine (BUP)

Buprenorphine, a partial µ-opioid receptor agonist, is commonly used as a preferred medication for opioid use disorder during pregnancy, in part because clinical evidence suggests it produces a less severe neonatal opioid withdrawal syndrome than methadone (Zhang et al., 2018). Its metabolism is dominated by CYP3A4-mediated N-dealkylation, with smaller contributions from CYP2C8, UGT1A1, UGT1A3, and UGT2B7 (van Hoogdalem et al., 2024; Soyka, 2013). Modeling efforts began with a PBPK framework developed by Zhang et al. in Simcyp (v15), in which a perfusion-limited sublingual model constructed for healthy nonpregnant adults was adapted for pregnancy (Abduljalil et al., 2020). The fetal–placental unit was represented as a single perfusion-limited compartment incorporating the fetus, placenta, amniotic fluid, uterus, and umbilical cord. Tissue partition coefficients were estimated using a corrected Poulin–Theil method. Pregnancy-related changes expected to influence pharmacokinetics such as altered hepatic blood flow, shifts in plasma protein binding, enzymatic induction, and changes in red blood cell–plasma partitioning, were incorporated into the model. Simulated buprenorphine maternal concentration time profiles aligned reasonably well with observed data in the second and third trimesters and the postpartum period, aside from a modest underprediction of average concentrations in the third trimester, with most predicted dose-normalized exposure metrics falling within ±25% of observed means.

Validation of this model was limited to maternal data from mid- and late-pregnancy. Since fetal or placental concentrations were not measured in the original clinical studies, fetal predictions could not be externally confirmed. An attempt by Silva et al. to replicate this model using the same platform reported clearance estimates approximately 30% lower than those originally described (Silva et al., 2022). This failure to reproduce might be due to incomplete documentation of the customized sublingual administration approach, which relied on a combination of inhalation route parameters and oral absorption components, and a “depot” release mechanism to mimic the complex sublingual process.

More recently, van Hoogdalem et al. developed a more structurally refined maternal–fetal PBPK model in Simcyp (v21.0) (van Hoogdalem et al., 2024). This model includes full compartment models for maternal and fetal systems connected by a permeability-limited placenta. Transplacental transfer was predicted by physicochemical properties and modeled as passive diffusion, consistent with the absence of evidence for transporter involvement. Volume of distribution was predicted by predicting *K*_p_ using the Rodgers–Rowland method with a single empirical scalar (0.26819) across all maternal and fetal tissues. This single scalar assumes proportional error across all maternal tissues and extends the same assumption to fetal tissues, even though gestation alters physiology heterogeneously. Standard pregnancy-associated enzyme induction profiles provided by the software were used for hepatic CYP3A4 and fetal enzymes. The authors assumed that hepatic CYP2C8 and intestinal CYP3A4 followed the same induction pattern across gestation as hepatic CYP3A4 and utilized the UGT1A1 induction pattern to reflect UGT2B7 changes with gestation.

Because Simcyp does not provide a dedicated buccal absorption model, sublingual dosing was approximated by repurposing the inhalation model coupled with first-order absorption. Buprenorphine’s sublingual absorption is dependent on saliva pH, which is lower in pregnancy. The model predicted a 24.6% reduction in bioavailability during pregnancy. Based on this assumption, the median maternal plasma-concentration of buprenorphine in the second and third trimesters and postpartum were comparable to observed data, with most exposure metrics falling within a 1.25-fold prediction error range. However, the model’s *a priori* simulations failed to accurately predict individual maternal plasma concentrations at delivery, generally underpredicting exposure, due to the high inter-individual variability of sublingual bioavailability (15.7%–94.4%). To resolve this, the authors utilized post hoc optimization to optimize the individual absorption fraction (ranging from 0.1% to 99.9%) until the simulation matched the observed maternal plasma concentrations. Accurate predictions of fetal exposure (95.2% agreement with cord blood data) were only achievable after this manual calibration. This approach confirmed the authors’ hypothesis that the high inter-individual variability in bioavailability (which ranges from 15.7% to 94.4% in literature) prevents the model from making accurate *a priori* predictions based on dose alone.

Across the PBPK models of buprenorphine, a consistent pattern emerges: pregnancy substantially increases buprenorphine clearance, producing a gestation-dependent reduction in systemic exposure, primarily due to CYP3A4 induction. This provides a mechanistic explanation for the clinical reports of withdrawal symptoms in pregnant patients on standard maintenance doses and supports the need for gestational age-adjusted dosing strategies. Additionally, van Hoogdalem et al.’s model indicates that while maternal exposure decreases, fetal concentrations were predicted to increase across gestation (15–40 weeks) secondary to increased placental surface area (van Hoogdalem et al., 2024). However, it should be noted that the model was only validated against cord blood concentrations collected at term.

### 3.2 Methadone

Methadone has served as a primary treatment for opioid use disorder during pregnancy. Yet the drug remains challenging to manage as maternal plasma concentrations vary widely between individuals, and dose escalation is frequently required as pregnancy progresses to prevent withdrawal (Holbrook, 2015; Ray-Griffith et al., 2019). Methadone is eliminated predominantly by hepatic metabolism through CYP2B6, CYP3A, and CYP2C19, with renal clearance contributing roughly 16%–27% (Chang et al., 2011; Kharasch et al., 2015). Two PBPK studies were identified that evaluated methadone disposition during pregnancy (Table 3). Both studies employed mechanistic PBPK frameworks to characterize maternal PK, incorporating pregnancy-related physiological changes. No PopPK models of methadone in pregnant populations were identified.

A 2013 PBPK model developed by Ke et al. in Simcyp (v12.1) represented methadone metabolism by explicitly incorporating pregnancy-related changes in CYP3A, CYP2B6 and CYP2C19. A two-fold increase in CYP3A activity in the second and third trimesters was assumed based on previous work with midazolam, nifedipine and indinavir. CYP2B6 induction was estimated using a bottom-up in vitro-in vivo extrapolation (IVIVE) approach based on estradiol-driven changes in CYP2B6 mRNA (Dickmann and Isoherranen, 2013), whereas CYP2C19 suppression was inferred using a top-down strategy that relied on PK data from proguanil (Ke et al., 2014a). Verification efforts showed that the expanded model captured methadone disposition in the second trimester well, with predicted/observed ratios falling inside the 0.80–1.25 range. Consistent with clinical practice, the model reproduced the increase in methadone clearance during pregnancy and produced exposure profiles that tracked closely with observed clinical data.

More recently, a 2021 PBPK study by Badhan et al. predicted both R- and S-methadone concentrations in maternal and fetal blood across gestation using Simcyp (v19) (Badhan and Gittins, 2021). Parameter refinement used extensive single- and multiple-dose data from nonpregnant subjects, leading to different first-order absorption and volume of distribution parameters for each stereoisomer. Scalars for intrinsic clearance were optimized as IVIVE methods did not adequately reproduce observed clearance in nonpregnant adults. While enantiomer-specific plasma concentrations of R- and S-methadone were available for development and validation of the model in nonpregnant adults and umbilical cord blood at delivery, predicted R- and S-methadone concentrations were only compared to racemic methadone concentrations in maternal plasma at 27- and 37- weeks’ gestation. Despite these limitations in model validation, the authors utilized the model to predict and dose-optimize R- and S-methadone across gestation (0–40 weeks) as well as the impact of CYP2B6 and CYP2C19 polymorphisms. While the authors describe the equation governing suppression of CYP2C19 activity across gestation, it appears that default Simcyp equations for pregnancy were employed for all other parameters. As pregnancy advanced, the model predicted the expected decline in maternal methadone concentrations. However, fetal exposure increased in the third trimester due to higher fetoplacental blood flow. The authors went further by proposing an “optimal” gestation-adjusted dosing schedule after their simulations showed that a fixed 90-mg daily dose performed poorly: at term, 75% of simulated subjects for R-methadone and 94% for S-methadone had trough concentrations below the therapeutic threshold. An escalated regimen of up to 180 mg per day in the third trimester reduced the proportion of individuals with subtherapeutic concentrations. The study also underscored the clinical relevance of CYP2B6 genotype, reporting that poor metabolizers exhibited substantially elevated maternal and fetal methadone levels and a higher proportion of subjects exceeded the S-methadone toxicity thresholds (C_max_ >600 ng/mL).

These two PBPK modeling efforts for methadone in pregnancy come with notable differences and limitations. A fundamental issue shared across studies is the need to back-calculate hepatic unbound intrinsic clearance values for R- and S-methadone, because standard IVIVE approaches were unable to recover observed clearance when relying solely on *in vitro* metabolic data. As there may be limited clinical data to inform various pathways, this may lead to incorrect assumptions in the contribution of individual enzymes to the total clearance of each enantiomer.

### 3.3 Fentanyl

Fentanyl is widely used as a potent synthetic opioid to mitigate labor-induced pain, often administered epidurally. Its narrow therapeutic index and potential for transplacental transfer pose a risk of neonatal side effects, such as respiratory depression. The primary challenge addressed by these studies is predicting drug exposure in vulnerable populations (pregnant women and newborns) where clinical data collection is scarce (de Barros Duarte et al., 2009; Kumar and Paes, 2003). Three studies reported PK modeling strategies for fentanyl in the obstetric population (Table 3). Cambic et al. employed a non-mechanistic compartmental model to evaluate the potential safety risks of ritonavir-induced CYP3A4 inhibition during patient-controlled epidural analgesia, aiming to determine if maternal plasma concentrations would reach thresholds for respiratory depression (Cambic et al., 2014). Taking a more mechanistic approach, Shum et al. developed a maternal–fetal (mf) PBPK model to predict fetal exposure following maternal epidural and intravenous administration, including simulations of high-dose illicit use (Shum et al., 2021). Alsmadi further advanced this PBPK framework to predict fentanyl concentrations in specific newborn tissues, aiming to link non-invasive sampling of saliva and urine to drug levels in the brain extracellular fluid (bECF), the pharmacodynamic target for respiratory depression (Alsmadi, 2023).

The study by Cambic et al. addressed a specific safety concern: the potential for ritonavir, a strong CYP3A4 inhibitor used in HIV therapy, to increase maternal fentanyl levels during patient-controlled epidural analgesia (Cambic et al., 2014). Their approach relied on a non-mechanistic compartmental PK model, used largely as a pragmatic tool to explore a clinically important drug–drug interaction in a population too small and ethically complex for prospective trials. The structural model employed a 3-compartment distribution model with absorption of fentanyl from the epidural space modeled using two parallel tanks-in-series structures (D_F(fast)_ and D_S(slow)_), reflecting fast and slow absorption components. Absorption parameters were estimated by fitting the model to fentanyl plasma concentrations observed in the first 20 min after epidural administration in laboring patients at term. However, the systemic disposition of fentanyl was described using the average parameters of the three-compartment PK model developed by Scott and Stanski (Scott and Stanski, 1987), which were derived from non-pregnant patients, an assumption that neglects pregnancy-related changes in cardiovascular physiology and hepatic clearance. The effect of ritonavir was incorporated as a 67% reduction in fentanyl clearance, based on a study in health volunteers (Olkkola et al., 1999). Using this model, the authors demonstrated that even under maximal dosing conditions, predicted maternal plasma concentrations (maximum 3.4 ng/mL) remained well below the ventilatory depression threshold derived from healthy male volunteers (EC_50_,MV = 6.1 ng/mL). However, the model has several limitations. It did not account for changes in CYP3A activity in pregnancy, which would impact both fentanyl and ritonavir clearance. Ritonavir’s effect was incorporated as a static covariate, which does not account for time- or dose-dependent exposure. There was also a lack of validation data for ritonavir or fentanyl concentrations in term laboring patients.

A more mechanistic approach was taken by Shum et al., who developed a mf-PBPK model in MATLAB and the Simulink platform (R2019b; MathWorks, Natick, MA) to predict maternal and fetal fentanyl exposure following intravenous and epidural administration, including misuse scenarios involving high-dose bolus injections (Shum et al., 2021). Their model consisted of 14 maternal compartments and 7 fetal compartments, linked by a placenta modeled as its own kinetic unit and parameterized for a term pregnancy (gestational week 40).

A key methodological innovation was the construction of an epidural dosing site model, which required assuming a permeability-limited absorption into epidural veins scaled from alfentanil because the actual surface area of the epidural venous plexus is unknown. The investigators also incorporated experimental evidence that CYP3A7, the predominant fetal CYP3A isoform, contributes to fentanyl metabolism in fetal liver microsomes, enabling estimation of fetal intrinsic clearance. Validation against clinical data showed that the model adequately captured observed maternal and umbilical cord concentrations after epidural dosing, with most absolute average fold errors staying within the acceptable ≤2-fold range. The model further predicted that large intravenous bolus doses could result in umbilical vein concentrations surpassing the reported EC50 for fentanyl, raising concerns about fetal safety following high-dose exposure. Importantly, the study demonstrated that commonly reported fetal concentration metrics at delivery, such as the umbilical vein–to–maternal vein (UV/MV) concentration ratio, are profoundly time-dependent and can be misleading due to delayed tissue distribution of drug; the UV/maternal artery ratio was recommended as a more reliable indicator of placental transfer. The main uncertainty affecting their fetal predictions was the poorly defined fraction unbound of fentanyl in umbilical cord plasma—an input that had a major influence on predicted fetal exposure.

The Alsmadi study approached the problem from a neonatal perspective, building a PBPK model to predict fentanyl concentrations in newborn tissues, particularly in brain extracellular fluid (bECF), after transplacental exposure from maternal epidural fentanyl dosing (Alsmadi, 2023). Their work is notable because it links neonatal plasma, saliva, and urine concentrations, matrices more feasible and ethically acceptable for sampling, to fentanyl levels at the pharmacodynamic target site in the brain. The modeling strategy involved first constructing and validating an adult PBPK model utilizing the PK-Sim/MoBi platform. They then adjusted physiological parameters to account for differences in pregnancy and in full-term and preterm neonates using system-specific maturation functions for metabolic enzymes, renal excretion, and transporter activity. The model incorporated CYP3A4/CYP3A7 metabolism, P-glycoprotein efflux at the blood–brain barrier, and a three-compartment epidural dosing space. Several assumptions were unavoidable, including the use of adult Vmax and Km values and adult partition coefficients for maternal and neonatal tissues, as well as assuming adult CYP3A7 activity in pregnancy due to a lack of published data. Model performance across adult and neonatal datasets generally met standard validation criteria (0.5–2 fold error). Predicted bECF concentrations were compared to observed concentrations in adults, indicating an average fold error of 0.22, with most bECF concentrations being under-predicted. The model indicated a near-perfect correlation between the AUCs of fentanyl in neonatal saliva and bECF, suggesting that saliva could serve as a practical surrogate for neonatal CNS exposure. Simulations demonstrated that neonatal exposure following typical maternal epidural doses (245 µg) would remain below adult respiratory toxicity thresholds. However, the estimated safety thresholds were based entirely on adult IC50 values, which may underestimate infant sensitivity to fentanyl’s respiratory effects.

Across these studies, several recurring limitations emerge. Many of the physiologic and pharmacodynamic parameters needed for modeling, such as metabolic rates, protein binding, and toxicity thresholds, are unknown or poorly characterized in pregnant women, fetuses, and newborns. Both the Cambic and Alsmadi models relied on adult metabolic parameters or tissue partition coefficients, a problematic assumption given the differences in drug disposition across gestation. All three studies ultimately depended on toxicity thresholds derived from adults, despite evidence that neonatal respiratory depression can occur at much lower plasma concentrations. Shum’s fetal predictions were particularly sensitive to assumptions about the fraction unbound of fentanyl in umbilical cord blood, a parameter for which reliable data are still lacking. Gaps in enzyme ontogeny data, such as the absence of pregnancy-specific CYP3A7 expression levels, further complicate the mechanistic fidelity of maternal–fetal PBPK models.

Methodological limitations also affect model robustness. The compartmental model used by Cambic et al. lacks the physiological resolution needed to account for gestational changes in perfusion or tissue distribution and provides no ability to evaluate individual variability or vulnerable subgroups. The assumption, common in clinical literature, that single-time-point cord blood ratios reflect steady-state transfer kinetics was shown by Shum et al. to be unreliable. PBPK representations of epidural dosing remain approximate, relying on assumptions about drug behavior at the injection site that cannot currently be validated with direct measurements.

Despite these challenges, these studies contribute meaningful insights into fentanyl disposition in pregnancy and early life, particularly regarding maternal–fetal transfer and neonatal exposure. The Shum and Alsmadi PBPK models provided quantitative validation metrics, AAFE, AFE, and fold-error analyses, that generally met accepted predictive accuracy thresholds. Cambic et al., though limited by structural simplicity and lack of pregnancy-associated changes in clearance, demonstrates the value of modeling and simulation in understanding clinical effect of drug interactions in a difficult-to-study population. Together, these modeling efforts underscore both the feasibility and the limitations of predicting fentanyl disposition in pregnant women and neonates, highlighting the need for more targeted physiological data, improved measurement of fetal binding characteristics, and clearer reporting standards to enhance reproducibility in future pharmacometric work.

### 3.4 Oxycodone

Oxycodone is an opioid used for acute pain management, including analgesia during labor (Kokki et al., 2012). Hydrocodone and oxycodone are highly prescribed during pregnancy (3.6% and 2.2% rates in the study sample, respectively), yet they lack adequate PK studies in the pregnant population (Shendre et al., 2025). In this setting the drug is administered over a short period, and the primary concerns center on achieving adequate maternal pain control while minimizing immediate neonatal exposure. Despite its widespread use, the PK of oxycodone during labor had not been well characterized, which is notable given the substantial physiological shifts of pregnancy and the additional hemodynamic and hormonal stress associated with labor itself—factors that can meaningfully alter drug disposition (Evron and Ezri, 2007; Seaton et al., 2007). Because oxycodone readily crosses the placenta, understanding fetal exposure is essential before making evidence-based recommendations for obstetric use (Kokki et al., 2012).

The prospective, open-label clinical study underpinning the PopPK model by Kokki et al. was designed to characterize the maternal PK of intravenous oxycodone administered during the first stage of labor, and neonatal exposure to both the parent drug and its primary metabolites: noroxycodone, noroxymorphone, and oxymorphone. The model’s development was driven by the need to describe oxycodone disposition in laboring women and to estimate how maternal concentrations at delivery relate to umbilical cord drug levels. PopPK analyses were conducted in NONMEM (version VI, level 2.0) using the first-order conditional estimation method with interaction (FOCE-I). A two-compartment disposition with first-order elimination adequately described the observed plasma concentration–time profiles. The final model produced parameter estimates for clearance (0.92 L/min), central volume of distribution (95 L), peripheral volume of distribution (100 L), and inter-compartmental clearance (2.45 L/min). The study found that the elimination half-life of intravenous oxycodone in laboring women was considerably shorter than reported for nonpregnant adults, with a median value of 2.6 h compared to 3.8 h. This is a result of both higher clearance and reduced volume of distribution in pregnancy as compared to nonpregnant populations. Maternal plasma concentrations measured at delivery showed a strong correlation with neonatal umbilical cord concentrations, suggesting that maternal levels might serve as a practical surrogate for estimating fetal exposure in clinical scenarios where direct fetal sampling is not feasible (Kokki et al., 2012).

Several limitations should be acknowledged. The most notable constraint stems from the relatively small sample size (n = 15), which limited the precision of between-subject variability estimates and increased uncertainty for covariate effects that could not be evaluated. The authors appropriately describe this as a pilot dataset and given the challenges of conducting PK studies during labor—where recruitment, sampling windows, and ethical constraints are all pronounced—the limited cohort is not surprising, though it undeniably affects generalizability and ability to assess effects of covariates, such as CYP2D6 phenotype. Despite these limitations, the study provides one of the few quantitative characterizations of oxycodone PK in laboring individuals and offers a foundation for future modeling efforts requiring more granular mechanistic detail and larger, genotype-stratified cohorts.

### 3.5 Codeine and morphine

Codeine is prescribed to 2.9% of the pregnant population but has fewer than five published PK studies (Shendre et al., 2025). A PBPK model developed in Simcyp (v19.1) by Badaoui et al. predicted the disposition of codeine and its active metabolite, morphine, throughout pregnancy, predicting a progressive decline in both Cmax and AUC as gestation advanced (Badaoui et al., 2021). The model integrated the default Sim-Pregnancy population module with a single fetoplacental unit to account for physiological changes across gestation. CYP2D6 phenotype categories were explicitly defined by adjusting enzyme abundance for poor (PM), extensive (EM), and ultra-rapid (UM) metabolizers, with population frequency set to 0.333 for each phenotype. The model also incorporated the default pregnancy-related increase in CYP2D6 abundance, specifically a doubling of expression during the third trimester, consistent with reported increases in CYP2D6 activity during gestation. Following codeine administration, the model predicted 1.65- to 1.87-fold higher fetal morphine AUC in the CYP2D6 ultra-rapid metabolizer group compared with extensive metabolizers, highlighting how maternal genotype may materially influence fetal exposure. Interestingly, despite higher maternal CYP2D6 activity as pregnancy progresses, the simulations suggested that the fetus may be at greatest risk of elevated morphine exposure in the first trimester, particularly between 6–12 weeks gestation. This counterintuitive trend was attributed to the opposing and dominant effect of increased maternal and fetal fluid volumes, which diluted drug concentrations more effectively than the increased metabolic clearance produced morphine. A primary limitation of this work is that the findings are derived from simulations in a virtual population; while the model was verified against non-pregnant data, there was no comparison to observed maternal or fetal concentrations in pregnancy.

### 3.6 Sufentanil

Sufentanil, a short-acting and highly potent opioid, is routinely used to provide analgesia during labor and delivery, (Le Guen et al., 2001; Cohen et al., 1996). A multicenter, prospective observational study addressed this gap by presenting a PopPK model of epidurally administered sufentanil in laboring women (Nie et al., 2025). The investigators sought to construct a quantitative framework capable of characterizing the time-dependent behavior of sufentanil and its placental transfer when co-infused with ropivacaine. Plasma concentrations of sufentanil were obtained from 41 women receiving a continuous epidural infusion of 0.1% ropivacaine and 0.3 μg/mL sufentanil at 0.5 and 1 h after the epidural loading dose, at delivery and 2 h after delivery. Sufentanil disposition after continuous epidural administration in laboring women followed a two-compartment pharmacokinetic model. Although detectable transplacental passage occurs, fetal exposure remains minimal: the intercompartmental clearance governing maternal-to-fetal transfer (CL_2_ = 0.0134 L/h, approximately 13,000-fold lower than maternal clearance) is exceptionally slow, and the umbilical cord distribution volume (V_2_ = 0.187 L) is negligible compared to the maternal central compartment (V_1_ = 519 L). This pharmacokinetic profile results in umbilical cord concentrations near or below the limit of detection (0.05–0.1 ng/mL) despite measurable maternal plasma levels (Nie et al., 2025). One notable prediction from the final model was a slow washout of placental sufentanil after epidural infusion cessation, suggesting that very high epidural doses should be avoided during labor to minimize neonatal exposure.

Limitations to the study include a relatively small dataset. Each participant contributed only a single umbilical blood sample collected at the time of cord clamping. This sparse sampling inevitably restricts the precision of population parameter estimation. The original authors noted that coadministration of ropivacaine introduces potential pharmacokinetic uncertainty; however, there is limited clinical or theoretical basis for a meaningful systemic PK interaction. Neither sufentanil nor ropivacaine is a significant CYP inhibitor or inducer, and epidural administration results in low systemic exposure for both agents (Borsuk et al., 2017; Bienert et al., 2025; Hansdottir et al., 1995). While a pharmacodynamic interaction, such as synergistic analgesia, is expected and clinically routine, a significant systemic PK interaction is highly improbable and likely does not compromise the validity of the model. With respect to placental transfer, sufentanil’s physicochemical properties presents a pharmacokinetic paradox: its high lipophilicity (octanol–buffer distribution coefficient of 1,737) (Nie et al., 2025; Mather, 1983)would typically favor passive diffusion across the placenta, yet transfer remains low because of the drug’s extensive protein binding (93%) and the relatively low maternal concentrations achieved through epidural administration. The final model estimated the intercompartmental clearance (CL2) governing movement from the maternal central compartment to the umbilical cord compartment as 0.0134 L/h, an exchange rate characterized as extremely slow relative to maternal systemic clearance (176 L/h), further reinforcing that fetal exposure under typical dosing conditions is minimal (CL2 representing 0.01% of maternal clearance).

## 4 Discussion

Pharmacometric modeling approaches, including PBPK and PopPK methods, are increasingly used to support the study of drug disposition in populations that are difficult to investigate using conventional designs, including pregnant individuals. In the context of opioid use during pregnancy, these approaches have begun to generate insights that cannot be readily obtained from noncompartmental or purely descriptive PK studies, particularly given the ethical and practical constraints on intensive sampling. Pharmacometric models have helped quantify the extent to which gestational physiological changes alter opioid disposition and have provided mechanistic explanations for longstanding clinical observations, such as increased dose requirements in late pregnancy and the occurrence of withdrawal symptoms in patients with opioid use disorder treated with standard regimens (Dallmann et al., 2018). Consistent with prior descriptive reviews, opioids commonly used for pain management during pregnancy, such as hydrocodone and oxycodone, remain underrepresented in pregnancy-specific PK studies despite widespread prescribing (Shendre et al., 2025). In contrast, medications used for opioid use disorder, including buprenorphine and methadone, as well as fentanyl used for labor analgesia, have been the focus of a greater number of pharmacometric investigations. Nevertheless, substantial gaps remain, with several clinically relevant opioids lacking any published PK models in pregnant populations.

A comparison of modeling strategies across studies highlights the varying strengths of PopPK and PBPK approaches (Table 1). PopPK studies typically rely on theoretical mathematical compartmental structures tailored to observed plasma drug concentration *versus* time data (Ke et al., 2014b; Ke et al., 2018). For buprenorphine, a two-compartment model adequately described absorption and elimination patterns, and pregnancy itself emerged as the covariate influencing clearance. However, recent modeling work by van Hoogdalem et al. demonstrates that the gestation-dependent reduction in systemic exposure is multifactorial, driven not only by increased maternal clearance via enzyme induction (CYP3A4, UGT) but also by substantially reduced sublingual bioavailability caused by pregnancy-induced decreases in salivary pH (Garrett and Chandran, 1985; Migliario et al., 2021; Jain and Kaur, 2015). Because buprenorphine is a weak base, the lower salivary pH increases its ionization, hindering passive diffusion across the oral mucosa (Garrett and Chandran, 1985; Laine et al., 1988; Mendelson et al., 1997; Weinberg et al., 1988). PBPK models, in contrast, take a mechanistic route by explicitly representing maternal organs, the placenta, and in more advanced versions, individual fetal tissues. Their development follows a consistent workflow: construct and verify a nonpregnant adult model, then apply gestation-specific physiological changes such as increased cardiac output and plasma volume, decreased albumin, and gestational age-dependent changes in metabolic pathways including CYP3A4, CYP2D6, CYP2B6, CYP2C19, and multiple UGT isoforms (van Hasselt et al., 2012). These changes reflect a clinical literature showing that pregnancy alters the disposition of drugs metabolized by these pathways, including compounds unrelated to opioids, such as the CYP2C19 probe proguanil (Ke et al., 2014a), underscoring the systemic nature of pregnancy-associated metabolic shifts. However, the specific mathematical functions used to describe physiological changes across gestation vary between models. It is important to note that many manuscripts, particularly those using the default population libraries within commercial software like Simcyp, often do not explicitly define these underlying parameters or the specific datasets used to derive them, which can obscure the exact mechanisms driving the predicted changes in drug disposition.

Placental transfer modeling represents one of the most technically challenging components of maternal–fetal PBPK models. Several of the reported opioid models used a combined fetoplacental compartment to approximate transfer kinetics (Table 3). More recent models, including the permeability-limited placenta published by van Hoogdalem et al., adopt a more mechanistic strategy that uses drug-specific physicochemical properties to estimate passive diffusion across the placenta. The most advanced structures seen, for example, in models of fentanyl disposition, link a full maternal PBPK model to a multi-compartment fetal model through a placental interface, enabling predictions of fetal tissue concentrations, including the fetal brain (Alsmadi, 2023; Shum et al., 2021). Although full maternal-fetal PBPK architecture is scientifically appealing, lack of fetal physiological and concentration data in individual tissues may limit model utility and validation.

Model evaluation practices show progress but remain variable across the literature. For PBPK models, the standard verification sequence begins with qualification in nonpregnant adults, followed by comparison of pregnancy-scaled predictions against sparse PK data from pregnant individuals, often limited to second or third trimester. Model performance is often assessed using visual overlays of predicted and observed concentration–time curves and fold-error comparisons of exposure metrics such as AUC and C_max_. Acceptance criteria in the opioid PBPK models allowed predictions within 0.5- to 2-fold of observed values, as compared to the more restrictive 0.8–1.25-fold criteria. PopPK studies rely more heavily on simulation-based diagnostics, with visual predictive checks used to evaluate model fit and nonparametric bootstrap analyses used to gauge parameter stability. While these methods are broadly accepted, they provide relatively limited insight into how uncertainty in gestational physiology propagates into PK predictions. As most datasets describing opioid PK in pregnancy are relatively small, there is limited ability to assess covariates in these models, limiting understanding of sources of inter- and intra-individual variability.

Reproducibility of models is critical for advancing the field yet remains a limitation across the reviewed studies. The failed attempt by Silva et al. to reproduce a previously published buprenorphine PBPK model illustrates how incomplete documentation can undermine the scientific utility of computational models (Silva et al., 2022). In that case, insufficient detail on the customized sublingual absorption model prevented replication of the healthy adult model. Similar concerns arise in models that employ empirically scaled tissue-partition coefficients without experimental justification. Applying a single global scalar assumes proportional error across all tissues and fails to capture the heterogeneous physiological alterations that occur during gestation. A more rigorous approach utilizes built-in mechanistic prediction algorithms (e.g., Rodgers and Rowland or Poulin and Theil methods) that calculate tissue-to-plasma partition coefficients using drug-specific physicochemical properties integrated with gestation-dependent tissue compositions. When implemented within platforms such as Simcyp, these mechanistic equations dynamically update partition coefficients as the virtual pregnancy progresses, avoiding the “locked” static values that result from manual overrides. If empirical optimization is required, it should be restricted to specific compartments with clear justification rather than applied globally across maternal and fetal tissues. While several manuscripts provided schematic model diagrams, none provided model codes or output files providing details on model inputs. These issues underscore a broader need for standardized reporting that mandates full parameter tables, explicit documentation of software inputs, and public access to model code.

Across all models evaluated, one of the clearest scientific gaps is the limited inclusion of active opioid metabolites. Although the systemic contribution of opioid metabolites varies due to factors such as blood-brain barrier permeability, certain metabolites are pharmacologically significant. Recent preclinical evidence demonstrates that *in utero* exposure to norbuprenorphine is pharmacologically active and implicated in neonatal opioid withdrawal severity (Griffin et al., 2019; Tobacyk et al., 2023) in preclinical models (where clinically relevant doses induced fetal opioid dependence with brain concentrations predicting withdrawal severity) and clinical studies (where umbilical cord tissue concentrations correlated with peak Finnegan scores and cumulative morphine treatment doses) (Rana et al., 2023), although the precise contribution of norbuprenorphine to the overall clinical effects of buprenorphine remains uncertain (FDA. Buprenorphine Hydrochoride, 2026). For morphine, the active metabolite morphine-6-glucuronide crosses the blood-brain barrier and contributes to analgesia and respiratory depression, whereas morphine-3-glucuronide does not (Klimas and Mikus, 2014). Methadone metabolism is stereoselective and depends substantially on CYP2B6 activity, which shows strong interindividual variability and gestational modulation (Gadel et al., 2015). Fentanyl likewise produces metabolites, although norfentanyl is generally considered pharmacologically inactive (Smith, 2009). The exclusion of metabolites may not severely compromise maternal PK predictions for the parent drug, but it substantially limits the ability to estimate fetal exposure and neonatal outcomes. By focusing solely on the parent compound, studies may underestimate the total fetal exposure to pharmacologically active species. Consequently, the omission of metabolite PK leaves a gap in our understanding of the potential long-term developmental outcomes for the neonate. Incorporating metabolite kinetics, supported by gestation-specific enzyme ontogeny data, is a necessary next step for generating clinically meaningful pharmacodynamic models.

Several limitations are endemic to the current state of maternal-fetal pharmacometric modeling and apply broadly across the reviewed studies. These include: (1) sparse fetal sampling limited to single-timepoint cord blood at delivery, precluding longitudinal fetal PK validation; (2) major uncertainty in fetal and umbilical unbound fractions, which fundamentally affects placental transfer predictions; (3) reliance on extrapolated adult metabolic parameters (Vmax, Km) and tissue partition coefficients due to lack of pregnancy-specific data; and (4) simplified fetal compartmentalization in most models. Recognizing these as field-wide constraints, rather than limitations of individual studies, is essential for guiding future research priorities.

Given these limitations, several priorities for future work are clear. First, standardized reporting guidelines, analogous to existing frameworks for PBPK model qualification, should be uniformly adopted to support reproducibility and cross-model comparison. Second, uncertainty quantification must be more deeply integrated into opioid modeling efforts. Most studies predict population averages, with limited assessment of variability. Conducting sensitivity analyses which take into consideration the covariate relationship between parameters, may improve understanding of potential range of exposures for a drug. Such sensitivity analyses are critical when biological data on given parameters are missing. Third, improved empirical data are essential. Longitudinal maternal PK studies across all trimesters, routine cord-blood sampling at delivery, opportunistic sampling during clinically indicated cordocentesis (to verify predicted fetal concentrations prior to term), and better characterization of placental and fetal metabolism would significantly strengthen model verification (Berry et al., 2013; Tanvisut et al., 2020). While many of the PBPK models for opioids predicted exposure in the first trimester, no observed data were available to support the predictions. Additionally, fetal concentrations can only be validated using cord blood concentrations obtained at delivery. Caution should be used when interpreting predictions from earlier trimesters of pregnancy. Finally, modeling efforts must expand beyond buprenorphine and methadone to include opioids with high clinical relevance, such as tramadol, hydrocodone, and oxycodone, where PK behavior is strongly influenced by gestational physiology yet remains insufficiently characterized.

Collectively, the available opioid pharmacometric models in pregnancy reveal both the promise and the limitations of current approaches. While they consistently demonstrate that pregnancy markedly alters opioid disposition, inconsistent reporting practices, gaps in physiological representation, and persistent reproducibility problems limit the translational value of these findings. Addressing these issues through methodological rigor, greater transparency, and expanded empirical data will be essential for developing models that can meaningfully guide safe and effective opioid therapy during pregnancy.

## 5 Best practices and research agenda: an academic perspective on best practices for model development and reporting

Insights from the available literature, alongside recommendations from regulatory and academic groups, point toward a set of best practices that should guide future PopPK and PBPK model development in pregnancy. These principles are aimed at improving transparency, strengthening reproducibility, and enhancing the clinical utility of these models, goals that remain inconsistently met in the current body of work (Riedmaier et al., 2020; Jayachandran et al., 2024; Eke et al., 2020; David et al., 2022).

From a reporting standpoint, every modeling study should begin with a clearly articulated objective, whether it is dose optimization, prediction of fetal exposure, evaluation of interindividual variability, or assessment of specific physiological mechanisms. Clarity of purpose helps establish the model’s intended scope and prevents overinterpretation of results. Equally important is explicit justification of all key assumptions; whether physiological, structural, or statistical. These assumptions are often the largest sources of uncertainty, yet they are frequently underreported or relegated to appendices, limiting the ability of other investigators to critically evaluate or reproduce the work.

For PopPK models, several concrete reporting standards warrant emphasis. Studies should provide a complete account of the underlying data, including study design, participant demographics, gestational age distribution, sampling strategy, and analytic quantification methods. These details shape the reliability of parameter estimates and the strength of covariate associations. The structural model, statistical model forms, estimation algorithm, and residual variability structures should be described in sufficient detail to permit replication, ideally with model code provided as Supplementary Material. Covariate selection procedures, whether based on stepwise testing, biological plausibility, or predefined criteria, must also be transparently reported. Finally, model evaluation should include the established suite of diagnostics: goodness-of-fit plots, simulation-based checks such as VPCs, and bootstrap analyses to assess precision and robustness (Chaphekar et al., 2020).

PBPK models require an even higher standard of documentation due to their complexity. Every PBPK study should clearly specify the modeling platform and version, describe the steps taken to develop and verify the nonpregnant adult model, and explain how gestational physiology was incorporated. Transparency of parameters is essential; each drug-specific and system-specific parameter should be accompanied by its source, a justification for its value, and, where applicable, a description of any assumptions or scaling factors applied. Model codes and detailed input parameters should be provided as Supplementary Material. Verification must rely on independent datasets, not those used in model calibration, and should include both graphical overlays of predicted *versus* observed profiles and quantitative comparisons of key PK metrics. Predefined acceptance criteria are critical for avoiding *post hoc* rationalization, and a generic two-fold error threshold may be inappropriate for all compounds or use cases. Sensitivity analyses should be included to identify the parameters that exert the strongest influence on model predictions, highlighting where future data collection would have the greatest impact (Silva et al., 2022; Chaphekar et al., 2021).

Adherence to these best practices is essential if PBPK and PopPK models are to transition from exploratory academic exercises to tools that meaningfully support clinical decision-making in obstetric populations (van Donge et al., 2020).

### 5.1 A research agenda for the future: integrated feto-maternal models and unstudied opioids

The patterns observed across the reviewed studies suggest a clear research agenda that could substantially advance the field over the coming decade. The most immediate priority is the development of more comprehensive PBPK models for methadone. While early work by Badhan and colleagues has laid the groundwork, current models remain too limited to reliably predict fetal exposure or capture the impact of pharmacogenetic variation, particularly the substantial influence of CYP2B6 polymorphisms on methadone disposition (Badhan and Gittins, 2021). Matching the sophistication seen in buprenorphine PBPK models should be considered an urgent goal, given the continued reliance on methadone as a frontline OUD agent.

A second priority is to broaden the modeling landscape to include opioids frequently used outside OUD contexts. Many pregnant individuals receive opioids for chronic pain or acute analgesia, yet drugs such as hydromorphone, hydrocodone, and tramadol remain understudied in pregnancy, with only limited PK data available at delivery. Systematic development of PopPK or PBPK models for these agents would fill a clinically important gap.

A third area for advancement involves increasing the biological realism and personalization of future maternal–fetal models. Refinement of the fetoplacental unit to incorporate active transport mechanisms, gestational changes in placental metabolism, and the progressive maturation of fetal metabolic pathways would allow more accurate predictions of fetal exposure. Integrating pharmacogenomic data, particularly variants affecting CYP2B6, CYP2D6, and UGT2B7, would help explain the wide interindividual variability observed in opioid PK and support movement toward precision dosing.

The final priority is to extend modeling efforts beyond PK. Existing models excel at predicting drug concentrations, but their clinical value will be greatly amplified when linked to pharmacodynamic outcomes. Developing integrated PK/PD frameworks that connect maternal and fetal exposure of parent opioids and active metabolites to clinically meaningful endpoints, such as maternal withdrawal control, analgesic adequacy, or neonatal opioid withdrawal severity, represents the logical next step. Achieving this will require acquiring paired PK data and rigorous phenotypic outcomes, ideally across multiple gestational stages. Models capable of predicting both exposure and clinical response would mark a major step toward genuinely individualized perinatal opioid therapy.

## 6 Conclusion

Taken together, the evidence reviewed in this systematic analysis shows that pharmacometric modeling provides mechanistic insight into opioid disposition during pregnancy, but additional work is necessary to fully predict exposure across gestation. Existing PopPK and PBPK models successfully capture major gestational shifts in PK, yet substantial gaps remain in reproducibility, physiological completeness, and the representation of fetal and neonatal outcomes. Methodological inconsistencies, particularly underreporting of model assumptions, insufficient verification, and lack of transparency, further limit the clinical relevance of many published models.

Progressing from exploratory modeling and towards models capable of informing clinical care will require consistent reporting standards, improved integration of mechanistic pathways, incorporation of genetic and developmental determinants of variability, expansion of modeling efforts beyond a small subset of opioids, and collection of clinical data at earlier gestational age. In particular, maternal-fetal models will need to move beyond exposure prediction alone and more explicitly link PK to clinically relevant maternal and neonatal outcomes.

Collectively, these findings suggest that pharmacometric modeling has the potential to meaningfully inform opioid therapy during pregnancy, provided that future efforts are supported by richer empirical data, greater methodological rigor, and transparent reporting practices. Addressing these needs will be essential for translating modeling advances into quantitative tools capable of supporting individualized and evidence-based perinatal care.
