# Supplementary material for: Pharmacometric modeling of opioid disposition in pregnancy: a systematic review of PopPK and PBPK approaches, model limitations, and future directions
Source: Front Pharmacol. 2026 Apr 20;17:1789714. doi: 10.3389/fphar.2026.1789714 (PMC13136160; doi:10.3389/fphar.2026.1789714)
Supplement: Supplementary file 1 [file Table1.docx]

**Supplementary Table 1. Summary of study design, methodology, and maternal demographics.**

|  | **Study, Publication year** | **Subgroup** | **Study Design** | **Country** | **Software** | **Drug** | **Patients (N)** | **Range GA (wk)** | **Age (y)** | **Height (cm)** | **Weight (kg)** |
| --- | --- | --- | --- | --- | --- | --- | --- | --- | --- | --- | --- |
| 1 | van Hoogdalem et al., 2024 (1) |  | PBPK | USA | Simcyp v21.0 | Buprenorphine | 39 | 22.0-33.9 | 18-45 | Not reported | Not reported |
| 2 | Zhang et al., 2018 (2) | PK-2 (2^nd^ trimester) | P BPK | USA | SimCyp V15.1 | BUP Sublingual 8 ±3.1 mg | 8 | 22.0±2.5 | 27.3±5 | Not reported | 73.7±7.6 |
|  |  | PK-3 (3rd trimester) | PBPK | USA | SimCyp V15.1 | BUP Sublingual 10±3.7 mg | 13 | 34.0±2.2 | 27.9±5.2 | Not reported | 74.1±8.3 |
|  |  | PK-P (postpartum) | PBPK | USA | SimCyp V15.1 | BUP Sublingual 8±0 mg | 13 |  | 28.6±5.6 | Not reported | 66.5±9.4 |
|  |  | PK-2 (2^nd^ trimester) | PBPK | USA | SimCyp V15.1 | 8mg BID | 4 | Not reported | Not reported | Not reported | Not reported |
|  |  | PK-3 (3rd trimester) | PBPK | USA | SimCyp V15.1 | 8mg BID | 4 | Not reported | Not reported | Not reported | Not reported |
|  |  | PK-P (postpartum) | PBPK | USA | SimCyp V15.1 | 8mg BID | 10 | Not reported | Not reported | Not reported | Not reported |
| 3 | Badhan et al, 2021 (3) | Pregnancy | PBPK | UK | SimCyp V19 | Methandone (0.5 mg/kg; 0.4 mg/kg) | 8 | 20-37 | 20-30 | Not reported | Not reported |
| 4 | Ban Ke et. 2013, (4) |  | PBPK | USA | Simcyp v12.1; Matlab v. 7.10 | Methadone (30 mg) | 9 | Not reported | Not reported | Not reported | Not reported |
| 5 | Cambic et al, 2014 (5) | Laboring Women | PKSimulation | USA | SAMII | Fentanyl | Not reported | 37-40 | Not reported | Not reported | Not reported |
| 6 | Shum et al., 2021 (6) | Pregnant women at term | Mf-PBPK | USA | MATLAB and Simulink | Fentanyl | Not reported | 37-40 | Not reported | Not reported | 75.2 |
| 7 | Alsmadi, 2023 (7) | Pregnant Mother | PBPK | Jordan | PK-Sim 9, MoBi 9, Prism 9.0 | Fentanyl | 68 | 10-21 | 27-34 | 157 -162 cm | 67-85 |
| 8 | Badaoui et al, 2020 (8) | 6 weeks | PBPK |  | SimCyp V19 | Codeine 30 mg | 250 | 6 | Not reported | Not reported | 65.7 |
|  |  | 12 weeks | PBPK |  | SimCyp V19 | Codeine 30 mg | 250 | 12 | Not reported | Not reported | 67.3 |
|  |  | 24 weeks | PBPK |  | SimCyp V19 | Codeine 30 mg | 250 | 24 | Not reported | Not reported | 73.0 |
|  |  | 36 weeks | PBPK |  | SimCyp V19 | Codeine 30 mg | 250 | 36 | Not reported | Not reported | 78.7 |
| 9 | Kokki et al., 2012 (9) | Nulliparous parturients (1st stage labor) | Prospective, open-label, controlled | Finland | NONMEM (version VI) | Intravenous Oxycodone | 15 (Maternal) | 38–43 (Median: 40) | 18–38 (Median: 29) | 150–178 (Median: 161) | 56–103 (Median: 82) |
| 10 | Nie et al., 2025 (10) | Laboring women(Primiparas) | Multicentric, prospective, observational study | People’s Republic of China | NONMEM (version 7.4.0) | Sufentanil (administered with 0.1% ropivacaine) | 41 |  | 27.2 ± 2.9 (Mean ± SD) |  | 70.2 ± 7.5 (Mean ± SD) |

**Supplementary Table 2. Pharmacokinetic parameters reported in the studies.**

|  | **Author** | **Pregnancy Subgroup** | **Drug + Dose** | **Cmax (ng/mL)** | **Tmax (h)** | **AUC (ng/mL/h)** |
| --- | --- | --- | --- | --- | --- | --- |
| 1 | Zhang et al.(2) | 2^nd^ Trimester | Buprenorphine 8mg SL (BID) | 3.80 ±1.14 | 1.00 ±3.75 | 15.38 ±14.81 (0 -12h) |
|  |  | 3^rd^ Trimester | Buprenorphine 8mg SL (BID) | 2.62 ±0.57 | 0.47 ±0.6 | 12.41±6.51 (0 -12h) |
|  |  | Postpartum | Buprenorphine 8mg SL (BID) | 5.40 ±2.46 | 0.91 ±3.6 | 27.93 ±20.18 (0 -12h) |
|  |  | 2^nd^ Trimester | Buprenorphine 8mg SL (BID) | 3.36 ±1.17 | 1.10 ±0.2 | 16.21 ±9.50(0 -12h) |
|  |  | 3^rd^ Trimester | Buprenorphine 8mg SL (BID) | 3.01 ±1.00 | 1.09 ±0.26 | 13.00 ±6.21(0 -12h) |
|  |  | Postpartum | Buprenorphine 8mg SL (BID) | 5.39 ±1.31 | 1.04 ±0.27 | 29.62 ±8.65(0 -12h) |
| 2 | Van Hoogdalem et al. (1) | Pregnant women | Buprenorphine 8mg SL | Validated against Zhang (Ratios fell within 1.25-fold error range) | Validated against Zhang (Ratios fell within 1.25-fold error range) | Validated against Zhang (Ratios fell within 1.25-fold error range) |
| 3 | Ban Ke et. (4) | 2^nd^ Trimester | Racemic Methadone (30 mg, QD) | 108.9 | Not reported | 1778.7 |
|  |  | 3^rd^ Trimester | Racemic Methadone (30 mg, QD) | 94.5 | Not reported | 1539.0 |
|  |  | Postpartum | Racemic Methadone (30 mg, QD) | 190.2 | Not reported | 3298.1 |
| 4 | Badhan et al. (3) | Week 27(Phase 1) | R-Methadone (0.5 mg/kg) | 97.2 (15.5) | 1.8 (0.2) | 1255(299) |
|  |  | Week 27(Phase 1) | R-Methadone (0.5 mg/kg) | 75.7 (13.6) | 1.8 (0.2) | 1070.7(231) |
|  |  | Week 37(Phase 2) | R-Methadone (0.4 mg/kg) | 90.4 (30.3) | 1.15 (0.17) | 998.8(398) |
|  |  | Week 37(Phase 2) | R-Methadone (0.4 mg/kg) | 84.8 (30.2) | 1.15 (0.17) | 921.2(321) |
| 5 | Cambic et al (5) | Laboring Patients (Control) | Group A: PCEA (16 µg/h infusion, no bolus) | 1.5 | 24 | 25.3 |
|  |  | Laboring Patients (Control) | Group F: PCEA (24 µg/h + 100 µg bolus) | 1.8 | 24 | 30.2 |
|  |  | Laboring Patients (Ritonavir Group) | Group A: PCEA (16 µg/h infusion, no bolus) | 2.9 | 24 | 40.8 |
|  |  | Laboring Patients (Ritonavir Group) | Group F: PCEA (24 µg/h + 100 µg bolus) | 3.4 | 24 | 48.5 |
| 6 | Shum et al. (6) | Pregnant Woman (Term) | IV Bolus 500 µg (Maternal Vein) | 3.9 | Not reported | Not reported |
|  |  | Pregnant Woman (Term) | IV Bolus 500 µg (Maternal Artery) | 150 | Not reported | Not reported |
| 7 | Alsmadi (7) | Pregnant Female (Epidural Bolus) | Epidural Bolus (Observed Validation Data) | 0.5 | Not reported | 0.5 *(AUC _0-t_)* |
|  |  | Pregnant Female (Epidural Bolus) | Epidural Bolus (Model Prediction) | 0.3 | 0.3 | *0.3 (AUC 0-t)* |
| 8 | Badaoui et al (8) | 6 wks | Morphine after Codeine 30 mg | 0.036 | Not reported | 0.088 |
|  |  | 12 wks | Morphine after Codeine 30 mg | 0.035 | Not reported | 0.085 |
|  |  | 24 wks | Morphine after Codeine 30 mg | 0.031 | Not reported | 0.077 |
|  |  | 36 wks | Morphine after Codeine 30 mg | 0.027 | Not reported | 0.068 |
|  |  | 6 wks | Codeine 30 mg | 244.6 | Not reported | 630.7 |
|  |  | 12 wks | Codeine 30 mg | 232.7 | Not reported | 600.6 |
|  |  | 24 wks | Codeine 30 mg | 205.7 | Not reported | 536.8 |
|  |  | 36 wks | Codeine 30 mg | 179.0 | Not reported | 474.8 |
| 9 | Kokki et al. (9) | Nulliparous parturients (1st stage labor) | IV Oxycodone 2–5 mg (median 5 mg) | 19 (Median) | Not reported |  |
| 10 | Nie et al. (10) | Laboring women | Sufentanil 45 ug | Not reported | Not reported | Not reported |

References

1. van Hoogdalem MW, Tanaka R, Abduljalil K, Johnson TN, Wexelblatt SL, Akinbi HT, et al. Forecasting Fetal Buprenorphine Exposure through Maternal-Fetal Physiologically Based Pharmacokinetic Modeling. Pharmaceutics. 2024;16(3).

2. Zhang H, Kalluri HV, Bastian JR, Chen H, Alshabi A, Caritis SN, et al. Gestational changes in buprenorphine exposure: A physiologically-based pharmacokinetic analysis. Br J Clin Pharmacol. 2018;84(9):2075–87.

3. RKS B, R G. Precision dosing of methadone during pregnancy: A pharmacokinetics virtual clinical trials study - PubMed. Journal of substance abuse treatment. 2021 Nov;130.

4. Ke AB, Nallani SC, Zhao P, Rostami-Hodjegan A, Unadkat JD. Expansion of a PBPK model to predict disposition in pregnant women of drugs cleared via multiple CYP enzymes, including CYP2B6, CYP2C9 and CYP2C19. Br J Clin Pharmacol. 2014;77(3):554–70.

5. CR C, MJ A, DK G, CA W. Effect of ritonavir-induced cytochrome P450 3A4 inhibition on plasma fentanyl concentrations during patient-controlled epidural labor analgesia: a pharmacokinetic simulation - PubMed. International journal of obstetric anesthesia. 2014 Feb;23(1).

6. Shum S, Shen DD, Isoherranen N. Predicting Maternal-Fetal Disposition of Fentanyl Following Intravenous and Epidural Administration Using Physiologically Based Pharmacokinetic Modeling. Drug Metab Dispos. 2021;49(11):1003–15.

7. Alsmadi MM. Evaluating the Pharmacokinetics of Fentanyl in the Brain Extracellular Fluid, Saliva, Urine, and Plasma of Newborns from Transplacental Exposure from Parturient Mothers Dosed with Epidural Fentanyl Utilizing PBPK Modeling. Eur J Drug Metab Pharmacokinet. 2023;48(5):567–86.

8. Badaoui S, Hopkins AM, Rodrigues AD, Miners JO, Sorich MJ, Rowland A. Application of Model Informed Precision Dosing to Address the Impact of Pregnancy Stage and CYP2D6 Phenotype on Foetal Morphine Exposure. AAPS J. 2021;23(1):15.

9. Kokki M, Franco MG, Raatikainen K, Valitalo P, Sankilampi U, Heinonen S, et al. Intravenous oxycodone for pain relief in the first stage of labour--maternal pharmacokinetics and neonatal exposure. Basic Clin Pharmacol Toxicol. 2012;111(3):182–8.

10. Nie Y, Sun X, Cao R, Tang S, Zhou Q, Zhou M, et al. Population Pharmacokinetic of Epidural Sufentanil in Labouring Women: A Multicentric, Prospective, Observational Study. Drug Des Devel Ther. 2025;19:971–80.
